# Trends, Influence Factors, and Doctor-Patient Perspectives of Web-Based Visits for Thyroid Surgery Clinical Care: Cross-Sectional Study

**DOI:** 10.2196/47912

**Published:** 2023-11-07

**Authors:** Xinyi Wang, Anping Su, Feng Liu, Yanping Gong, Tao Wei, Rixiang Gong, Jingqiang Zhu, Zhihui Li, Jianyong Lei

**Affiliations:** 1 Division of Thyroid Surgery, Department of General Surgery West China Hospital Sichuan University Chengdu, Sichuan China

**Keywords:** internet hospital, outpatient, telemedicine, thyroid surgery, web-based visit

## Abstract

**Background:**

In recent years, the new generation of telecommunication technologies has profoundly changed the traditional medical industry. To alleviate the medical difficulties faced by patients with thyroid diseases, hospitals have opened web-based visits and actively combined online-to-offline outpatient services.

**Objective:**

This study aims to explore differences between office and web-based outpatient services from doctors’ and patients’ perspectives, illustrate the effect of the COVID-19 pandemic on outpatient services, and provide clues for improving the online-to-offline mode of care for patients with thyroid diseases.

**Methods:**

We collected the complete web-based and office outpatient records of the Thyroid Surgery Center of West China Hospital. A total of 300,884 completed patient encounters occurred (201,840 office visits and 99,044 web-based visits) from January 1, 2019, to May 31, 2022. We performed logistic regression to evaluate the association between the chosen visit type and patients’ sociodemographic characteristics.

**Results:**

The number of web-based visits rapidly increased since March 2020 and reached 45.1% (4752/10,531) of all encounters in December 2021. The COVID-19 pandemic dramatically accelerated the development of web-based visits. Web-based visits were preferred by patients 18-45 years old (odds ratio [OR] 2.043, 95% CI 1.635-2.552, *P*<.001), patients with relatively high-paying jobs (technical staff: OR 1.278, 95% CI 1.088-1.479, *P*=.003; office clerk: OR 1.25, 95% CI 1.07-1.461, *P*=.005; national public servant: OR:1.248, 95% CI 1.042-1.494, *P*=.02), and patients living in Sichuan Province (excluding Chengdu; OR 1.167, 95% CI 1.107-1.23, *P*<.001). The medicine cost (*P*<.001) and examination cost (*P*<.001) of office visits were significantly higher than those of web-based visits.

**Conclusions:**

Web-based outpatient visits have increased rapidly in recent years, and the COVID-19 pandemic has boosted their development. The preference for web-based visits was influenced by the socioeconomic and demographic characteristics of both patients and doctors.

## Introduction

Telemedicine is emerging as a novel method for doctors to provide medical services via telecommunication technologies [1,2]. Internet hospitals are medical platforms with consulting, follow-up, and chronic disease management functions, and they generally have the strong support of physical hospitals and provide web-based convenience for patients [3,4]. In fact, a combination of web-based and office visits is required to meet the diverse needs of patients. The online-to-offline clinical care model combines offline outpatient visits with the internet as a platform for web-based transactions [5,6]. Doctors who are registered in internet hospitals can perform diagnosis and treatment activities on the internet and provide succeeding services, including web-based consultation, prescribing, dispensing, and hospitalization registrations [7]. Web-based visits can be achieved at a distance, limiting time and money costs and providing continuing services for patients, and were advocated for in the context of the management of patients in central medicine departments [8]. Since December 2019, the COVID-19 pandemic has caused a sudden and profound conversion in medical activities [9]. Compulsory stay-at-home orders, mitigation strategies, and overloading of medical institutions have made normal health care impossible [7]. In response, health care systems quickly restricted in-person visits and procedures and expanded telemedicine services [10]. In April 2020, a few weeks after the shelter-in-place order was issued in the United States, Madduri et al [11] performed a web-based survey of endocrinologists who were members of the Facebook group “Endocrinologists” and illustrated the importance of web-based outpatient services. As the pandemic lessens, it is becoming obvious that telemedicine will continue to play some roles in routine health care [12]. Although the experience of health care providers regarding telemedicine during the pandemic has been reported, there are few studies on the experience of telemedicine for patients with specific diseases [13].

In the past, telemedicine was relatively limited to specific fields, such as psychiatry, primary care, and diabetes counselling, primarily providing health care for remote patients [14-18]. An otolaryngology pilot program using web-based outpatient services has gained high patient satisfaction, elevated numbers of accesses, and effective cost savings [19]. Surgeons are generally slow to adopt web-based outpatient services due to the substantial reliance on physical exams and specific instruments and diagnostic procedures [20]. Thyroid and parathyroid diseases may be uniquely well-suited to telemedicine [21]. Previous studies have demonstrated the possibility of web-based visits for serving postoperative thyroid and parathyroid patients, as such visits only involve wound inspection and discussion of pathology results [22,23]. Although a complete history and physical examination remain the basis of preoperative assessment, many surgical thyroid and parathyroid cases rely more on imaging and laboratory evaluation than on physical examinations [24]. Moreover, surgical management of thyroid and parathyroid diseases has trended toward referrals to high-volume hospitals, which can lead to challenges for many patients [25]. Nevertheless, there is a lack of knowledge on the implications of telemedicine in the surgical management of patients with thyroid diseases.

This study aims to find out differences between office and web-based outpatient services from both doctors’ and patients’ perspectives, explain the influence of the COVID-19 pandemic on the choice of outpatient service, and provide clues for improving the online-to-offline mode of care for patients with thyroid diseases.

## Methods

### Study Design

We examined outpatient visits in West China Hospital, a large integrated medical system with over 1.31 million members that uses a comprehensive outpatient-inpatient electronic health record (EHR) and patient portal (mobile apps). Web-based visits have been used widely in the clinic since January 1, 2019. Patients who schedule an outpatient appointment through the patient portal can choose between an office or web-based visit type. The doctor and schedule availability were comparable across visit types. The EHRs of web-based and office visits are interoperable and can be reviewed in either context. In both web-based and office visits, doctors had complete access to patients’ EHRs and documented web-based visit records directly through their EHR. In web-based visits, patients have a conversation via text and pictures with the doctor using the Huayitong app. Patients can upload test results and have their doctors advise them on treatments and prescribe medication or further tests.

### Setting

We extracted outpatient visit records from January 1, 2019, to May 31, 2022, and collected several patient characteristics, including patient sociodemographic characteristics, diagnosis, and treatment information. Office visit records were obtained from outpatient medical records (including doctor-written case records and EHRs). Web-based visit records were obtained from the Huayitong app medical record (including chat record and EHR). COVID-19–related data were obtained from the official website of the National Health Commission of the People’s Republic of China and the Health Commission of Sichuan Province [26,27].

### Participants

Patients who had completed one or more web-based or office outpatient visits with a registered doctor from the Thyroid Surgery Center of West China Hospital were enrolled in this study. For each patient included in this study, we used their EHR to identify patient sociodemographic characteristics (age, sex, nationality, marriage status, job, and residence). We also obtained their diagnosis and treatment (visit type, appointment mode, time of application, medicine and inspection cost, and surgery appointment) information from the information department of the West China Hospital. In total, 16 doctors from the Thyroid Surgery Center of West China Hospital were enrolled in this study; all of them offered office visits, and 13 of them also offered web-based visits. The basic information for these doctors is shown in Multimedia Appendix 1, including age, professional title, and telemedicine reception time. The proportion of patients who completed a visit with an individual doctor, the number of patient visit applications, and the number of doctor acceptances in a given time period were recorded.

### Statistical Analysis

Logistic regression was used to evaluate the association (odds ratio, OR) between visit types and patients’ sociodemographic characteristics (age, sex, nationality, marriage status, job, residence, and visit type). The outcome was set as visit type, with office visit set as the reference visit type. The independent variables included age (<18, 18-45, 45-65, or >65 years), sex (male or female), nationality (Chinese Han or ethnic minority), marriage status (unmarried, married, divorced, or widowed), job (liberal profession, technical staff, office clerk, retiree, national public servant, laborer, self-employed, or student), residence (Chengdu city; Sichuan Province, excluding Chengdu; or outside of Sichuan), and visit number (first visit or subsequent visit). A liberal profession was defined as a temporary, part-time job performed independently by an individual, usually on a project basis, without a permanent employer. Ethnic minorities included Zhuang, Manchu, Hui, Miao, Uyghur, Tujia, Yi, Mongolian, Tibetan, Buyi, Dong, Yao, Korean, Bai, Hani, Kazak, Li, Dai, She, Lisu, Gelao, Dongxiang, Gaoshan, Lahu, Shui, Wa, Naxi, Qiang, Tu, Mulao, Xibe, Kirgiz, Daur Jingpo, Maonan, Salar, Bulang, Tajik, Achang, Pumi, Ewenki, Nu, Jing, Jino, Deang, Baoan, Russian, Yugur, Wuziki, Menba, Oroqen, Dulong, Tatar, Hezhen, and Lhoba. The variables that showed a statistical difference were added as independent variables in the logistic regression analysis. Forward regression was implemented to reach the final model. The socioeconomic and demographic information of web-based and office patients were analyzed using a chi-square test. The medicine cost and inspection cost of office and web-based visits were compared using the Mann-Whitney test. Statistical significance was determined by 2-sided test, with *P*<.05 considered significant. The statistical analyses were performed using SPSS 26.0.0.0 (IBM) and R 4.2.0 (R Foundation for Statistical Computing). SEs were adjusted for the repeat visits using Stata version 14.2 (StataCorp LLC). We used GraphPad Prism 8 software (GraphPad) for graphical depictions.

### Ethics Considerations

The Institutional Review Board of the West China Hospital approved the research protocol and waived the requirement for written informed consent for participants in this data-only study (ethics approval number 2022-899; Multimedia Appendix 2).

## Results

### Socioeconomic Characteristics of Office and Web-Based Outpatient Services

**Figure 1:**
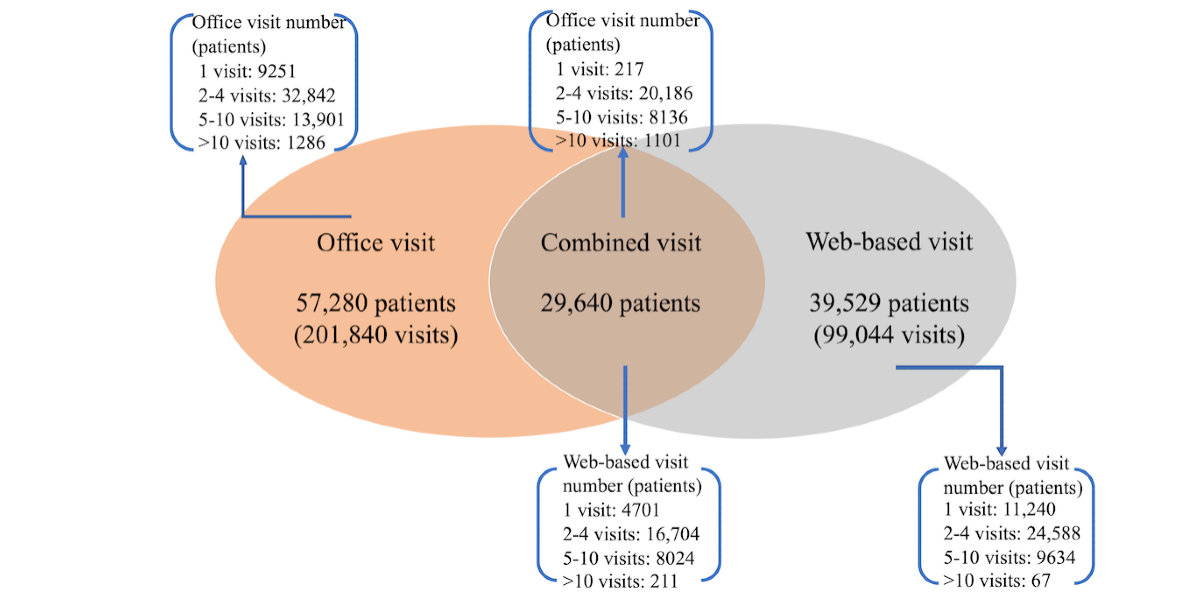
Overview of the number of office and web-based visits.

From January 1, 2019, to May 31, 2022, a total of 300,884 completed patient encounters occurred, of which 201,840 (67.1%) and 99,044 (32.9%) were office and web-based visits, respectively. Table 1 summarizes the characteristics of these patients. In total, 67,169 patients constituted all the encounters in this period, of which 51,379 (76.5%) had >1 encounter. Of the 300,884 patient encounters, the median age among all patients was 44 years (IQR: 42.5-44.9), 239,710 (79.7%) were female, 235,818 (78.4%) were of Chinese Han nationality, 9523 (3.2%) were considered an ethnic minority, and 59,283 (19.7%) did not provide their nationality. The highest proportion of thyroid diseases were of the thyroid nodules, affecting 85,076 of 201,840 (42.2%) office visits and 24,687 of 99,044 (24.9%) web-based visits. Except for nationality, there were statistically significant differences between office and web-based visits regarding age, sex, marital status, job, diagnosis, residence, and visit number. By encounter type, 39,529 of 67,168 (58.9%) patients had at least 1 web-based visit, and the remaining 27,640 (41.1%) patients had office visits only (Figure 1). The number of patients who completed 1, 2-4, 5-10, and >10 visits is also shown in Figure 1.

**Table 1:** Patient characteristics by visit type classification.

Characteristic		Office visit (n=201,840), n (%)	Web-based visit (n=99,044), n (%)		*P* value
**Age (years)**					
	<18	1777 (0.9)		708 (0.7)	<.001
	18-45	100,884 (50)		64,859 (65.5)	
	45-65	87,100 (43.2)		30,675 (31)	
	>65	11,802 (5.9)		2775 (2.8)	
	Not provided	277 (0.1)		27 (0)	
**Gender**					
	Male	42,982 (21.3)		18,999 (19.2)	<.001
	Female	158,666 (78.6)		80,044 (80.8)	
	Not provided	192 (0.1)		1 (0)	
**Nationality**					
	Chinese Han	156,386 (77.5)		79,432 (80.2)	.49
	Ethnic minority	5783 (2.9)		3740 (3.8)	
	Not provided	39,671 (19.7)		19,612 (19.8)	
**Marital status**					
	Married	97,723 (48.4)		25,601 (25.9)	<.001
	Unmarried	11,869 (5.9)		3915 (4)	
	Divorced	2958 (1.5)		768 (0.8)	
	Widowed	1244 (0.6)		272 (0.3)	
	Not provided	88,046 (43.6)		68,488 (69.1)	
**Job**					
	Liberal profession	10,724 (5.3)		5970 (6)	<.001
	Technical staff	22,723 (11.3)		15,576 (15.7)	
	Office clerk	34,918 (17.3)		22,455 (22.7)	
	Retiree	22,860 (11.3)		7829 (7.9)	
	National public servant	14,237 (7.1)		7915 (7.9)	
	Laborer	6136 (3)		2925 (3)	
	Self-employed	5674 (2.8)		3325 (3.4)	
	Student	3572 (1.8)		1996 (2)	
	Not provided	80,996 (40.1)		31,053 (31.4)	
**Diagnosis**					
	Thyroid cancer	28,178 (14)		12,351 (12.5)	<.001
	Postoperative re-examination	39,569 (19.6)		3165 (3.2)	
	Thyroid nodule	85,076 (42.2)		24,678 (25)	
	Parathyroid disease	939 (0.5)		4066 (4.1)	
	Hyperthyroidism	2819 (1.4)		1541 (1.6)	
	Hypothyroidism	6709 (3.3)		2274 (2.3)	
	Health examination	2319 (1.2)		434 (0.5)	
	Not provided	36,231 (18)		50,535 (51)	
**Residence**					
	Chengdu	106,266 (52.7)		51,376 (51.9)	<.001
	Sichuan (excluding Chengdu)	66,133 (32.8)		33,586 (34)	
	Outside of Sichuan	29,441 (14.6)		14,082 (14.2)	
**Visit number**					
	First visit	68,789 (34.1)		48,650 (49.1)	<.001
	Subsequent visit	132,751 (65.8)		49,403 (49.9)	
	Not provided	300 (0.1)		124 (0.1)	

### Increase in Web-Based Visits and Relationship With the COVID-19 Pandemic

**Figure 2:**
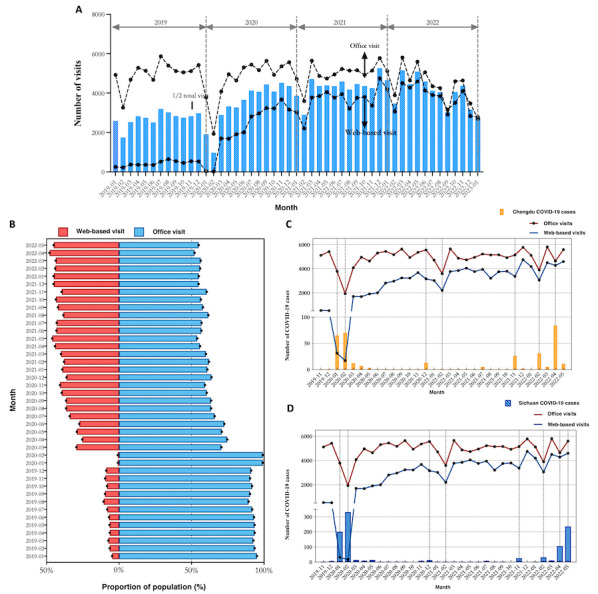
The development of outpatient services and their relationship with the COVID-19 pandemic. (A) The numbers of office and web-based visits from January 2019 to May 2022. (B) The proportions of patients participating in office and web-based visits. (C) The relationship between the number of outpatient visits and confirmed COVID-19 cases in Chengdu city. (D) The relationship between the number of outpatient visits and confirmed COVID-19 cases in Sichuan Province.

The number of office visits was relatively smooth and steady, constituting >90% of patient encounters before March 2020. It ranged from 4000 to 6000 visits in every month except for February, possibly due to the fewer number of days in February and the occurrence of the Chinese Spring Festival (Figure 2A). The number of web-based visits dramatically increased as of March 2020, with a peak in December 2021, during which time web-based visits constituted 4752 of 10,531 (45.1%) encounters (Figure 2B). Since December 2021, the proportion of web-based visits has remained over 40% but still lower than that of office visits. Chengdu city and Sichuan Province experienced several epidemic outbreaks of COVID-19, which greatly influenced the clinical activity of West China Hospital. As shown in Figure 2C and D, both office and web-based visits sharply decreased during the COVID-19 outbreak (ie, in January 2019, February 2019, December 2020, November 2021, February 2022, and April 2022). Especially in April and May 2022, the number of confirmed COVID-19 cases in Chengdu increased, which led to decreases in both office and web-based visits. However, the increase in confirmed COVID-19 cases in Sichuan province occurred 1 month later than that in Chengdu, indicating that outpatient services were mainly influenced by the epidemic situation in Chengdu.

### Demographic Characteristics of Office and Web-Based Outpatient Services

**Figure 3:**
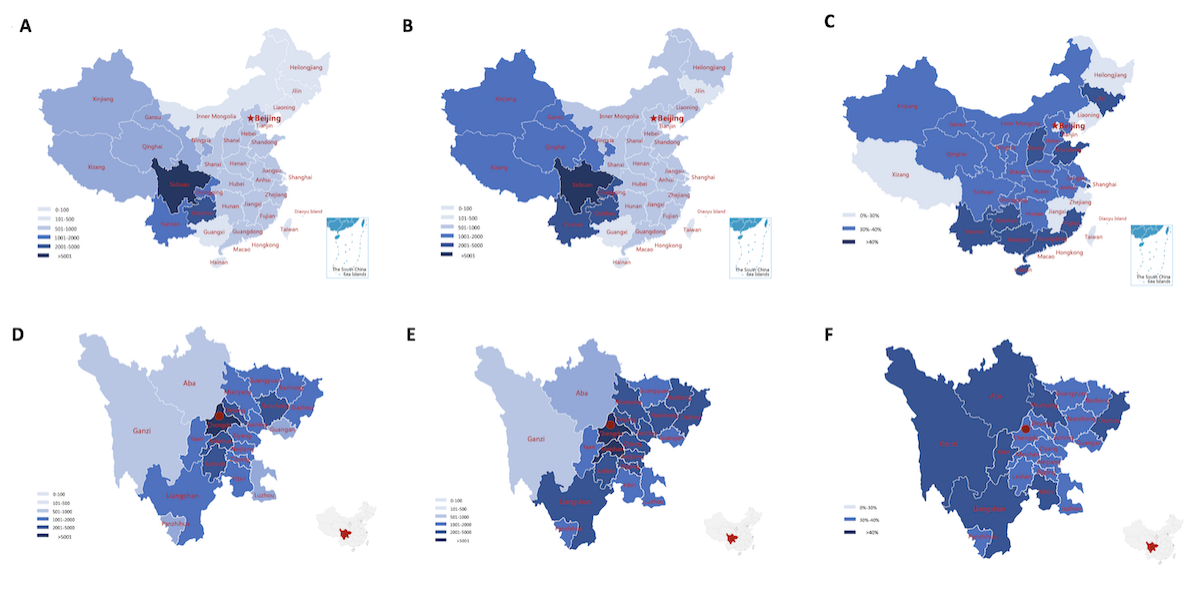
The geographical distribution of office and web-based patients. (A) The distribution of web-based patients in China. (B) The distribution of office patients in China. (C) The proportion of patients (%) participating in web-based visits in all provinces of China. (D) The distribution of web-based patients in Sichuan Province. (E) The distribution of office patients in Sichuan Province. (F) The proportion of patients (%) participating in web-based visits in all cities of Sichuan Province. A higher resolution version of the figure can be found in Multimedia Appendix 3.

Located in southwest China, the West China Hospital is a national center for the diagnosis and treatment of critical diseases. By analyzing the residences of patients, we found that the number of patients who lived in western China was dramatically higher than that in eastern China. The top 4 residences were Sichuan, Guizhou, Chongqing, and Yunnan. Both web-based visits (Figure 3A) and office visits (Figure 3B) presented this trend. For web-based visits, the number of encounters in Inner Mongolia, Liaoning, Jilin, and Heilongjiang was lower than those in other eastern provinces, which may be due to the relatively backwards economy and long distance. Figure 3C demonstrates that the proportion of web-based visits in southern provinces was higher than that in northern provinces. Similarly, we further analyzed the data of patients from cities in Sichuan Province and found that there were fewer patients from Ganzi and Aba than from other cities. However, the proportion of web-based visits in Ganzi and Aba was higher than those in other cities, indicating that web-based visits may provide convenience for patients who live in remote places.

**Figure 4:**
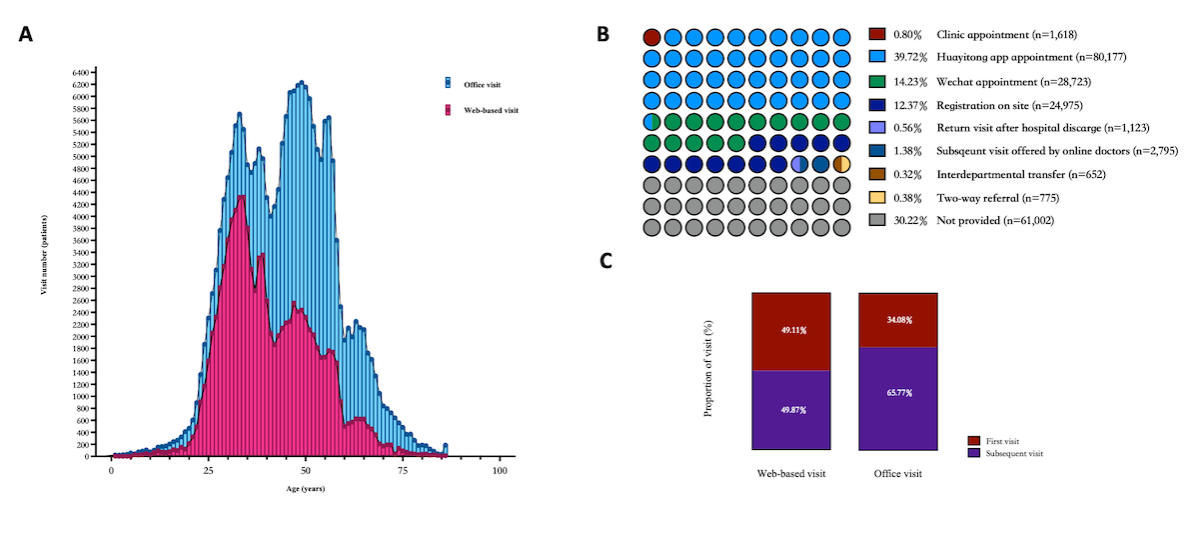
Characteristics of office and web-based patients. (A) The age distribution of office and web-based patients. (B) The proportion of office visit appointments made using different methods (n=201,840). (C) The visit number distribution of office and web-based patients.

The patients in the office visit and web-based visit groups also showed differences in age. As shown in Figure 4A, the highest frequency of web-based visits was among 34-year-old patients, while the highest frequency of office visits was among 51-year-old patients. The proportions of patients who were 18-45 years old were 100,884 of 201,840 (50.0%) for office visits and 64,859 of 99,044 (65.5%) for web-based visits, indicating that more young people preferred to choose web-based visits for their attendance (Table 1). The proportions of patients who were >45 years old were higher for office visits than for web-based visits, indicating that middle-aged and older individuals preferred to choose the traditional visit type.

There are several ways for patients to register office visits, including clinical appointments, Huayitong app appointments, WeChat appointments, registration on site, return visits after hospital discharge, subsequent visits offered by web-based doctors, interdepartmental transfers, and 2-way transfers (Figure 4B). It is worth noting that 53,483 of 201,840 (26.5%) patients registered office visits by telephone (ie, Huayitong app appointment or WeChat appointment). After the web-based visit, 2795 (1.4%) patients received a subsequent office visit offered by the doctor and did not need to register again. For web-based visits, 48,650 of 99,044 (49.1%) were the first visit for the patient, while 69,789 of 201,840 (34.1%) of office visits were first visits (Figure 4C). The proportion of visits on the weekend was dramatically higher for web-based visits (n=19,224; 19.4%) than for office visit (n=4585; 2.3%; Table 2). This demonstrated that more patients preferred to choose office visits for subsequent visits.

**Table 2:** The number of outpatient visits by weekday.

Weekday	Office visits (n=201,840), n (%)	Web-based visits (n=99,044), n (%)
Monday	36,736 (18.2)	12,277 (12.4)
Tuesday	53,645 (26.5)	13,814 (13.9)
Wednesday	43,357 (21.5)	13,789 (13.9)
Thursday	33,393 (16.5)	13,421 (13.6)
Friday	28,057 (13.9)	12,089 (12.2)
Saturday	2340 (1.2)	10,456 (10.6)
Sunday	2245 (1.1)	8768 (8.9)
Not provided	4417 (2.1)	11,430 (11.5)

### Factors Influencing the Choice of Visit Type

**Figure 5:**
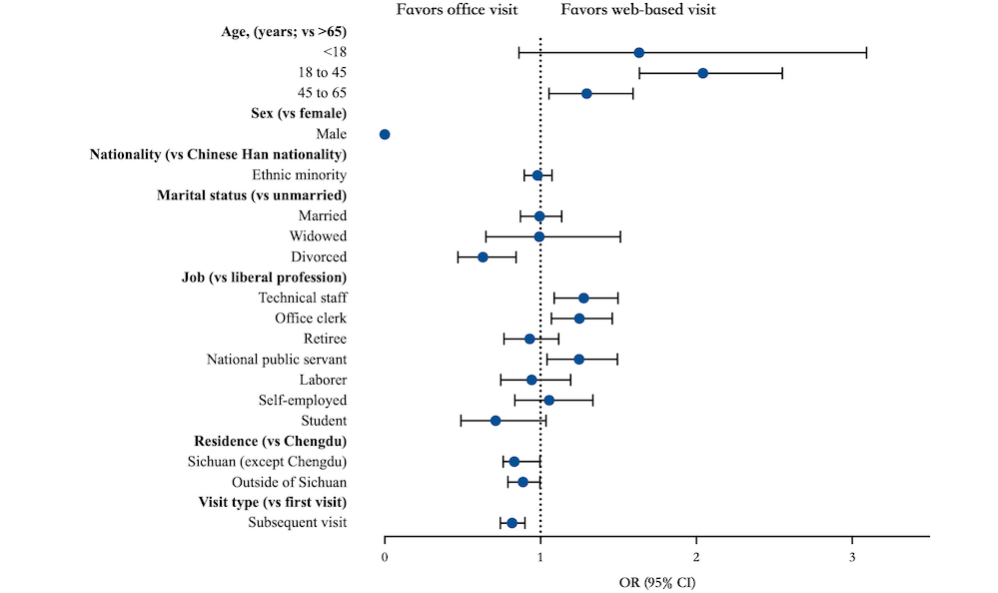
Logistic regression for the socioeconomic and demographic characteristics of patients by outpatient visit type.

To investigate factors that influence the choice of visit type, we performed a logistic regression analysis. The adjusted association between patients’ sociodemographic characteristics and outpatient visit type are presented in Figure 5 and Table 3. The results show that patients aged 18-45 years are 2.04 times more likely to choose a web-based visit than are patients older than 65 years (OR 2.043, 95% CI 1.635-2.552, *P*<.001). Also, patients aged 45-65 years are 1.30 times more likely to choose a web-based visit than are individuals older than 65 years (OR 1.297, 95% CI 1.055-1.594, *P*=.01). Compared to patients with liberal professions, those with positions as technical staff (OR 1.278, 95% CI 1.088-1.479], *P*=.003), office clerks (OR 1.25, 95% CI 1.07-1.461, *P*=.005), and national public servants (OR 1.248, 95% CI 1.042-1.494, *P*=.02) are 1.28 times, 1.25 times, and 1.25 times more likely, respectively, to choose a web-based visit. Patients living in Sichuan Province (excluding Chengdu) are 1.17 times more likely to choose a web-based visit (OR 1.167, 95% CI 1.107-1.23, *P*<.001). The probability of patients choosing a web-based visit for subsequent visits was lower than for the first visit (OR 0.818, 95% CI 0.743-0.9, *P*<.001).

**Table 3:** Logistic regression analysis of patient characteristics.

Characteristic		*P* value		Odds ratio (95% CI)
**Age (years; vs >65)**				** **
	<18	.13	1.633 (0.862-3.092)	
	18-45	<.001	2.043 (1.635-2.552)	
	45-65	.01	1.297 (1.055-1.594)	
**Sex (vs female)**				
	Male	.96	0.00 (0.00-4.49 × 10^267^)	
**Nationality (vs Chinese Han)**				
	Ethnic minority	.57	0.981 (0.896-1.074)	
**Marital status (vs unmarried)**				
	Married	.93	0.994(0.871-1.135)	
	Widowed	.97	0.63 (0.47-0.844)	
	Divorced	.002	0.992 (0.65-1.513)	
**Job (vs liberal profession)**				
	Technical staff	.003	1.278 (1.088-1.497)	
	Office clerk	.005	1.25 (1.07-1.461)	
	Retiree	.44	0.931 (0.766-1.116)	
	National public servant	.02	1.248 (1.042-1.494)	
	Laborer	.63	0.943 (0.745-1.193)	
	Self-employed	.65	1.056 (0.835-1.336)	
	Student	.08	0.712 (0.489-1.036)	
**Residence (vs Chengdu)**				
	Sichuan (except Chengdu)	<.001	1.167 (1.107-1.23)	
	Outside of Sichuan	.04	0.888 (0.791-0.996)	
**Visit type (vs first visit)**				
	Subsequent visit	<.001	0.818 (0.743-0.9)	

### Characteristics of Doctors Providing Outpatient Services

**Figure 6:**
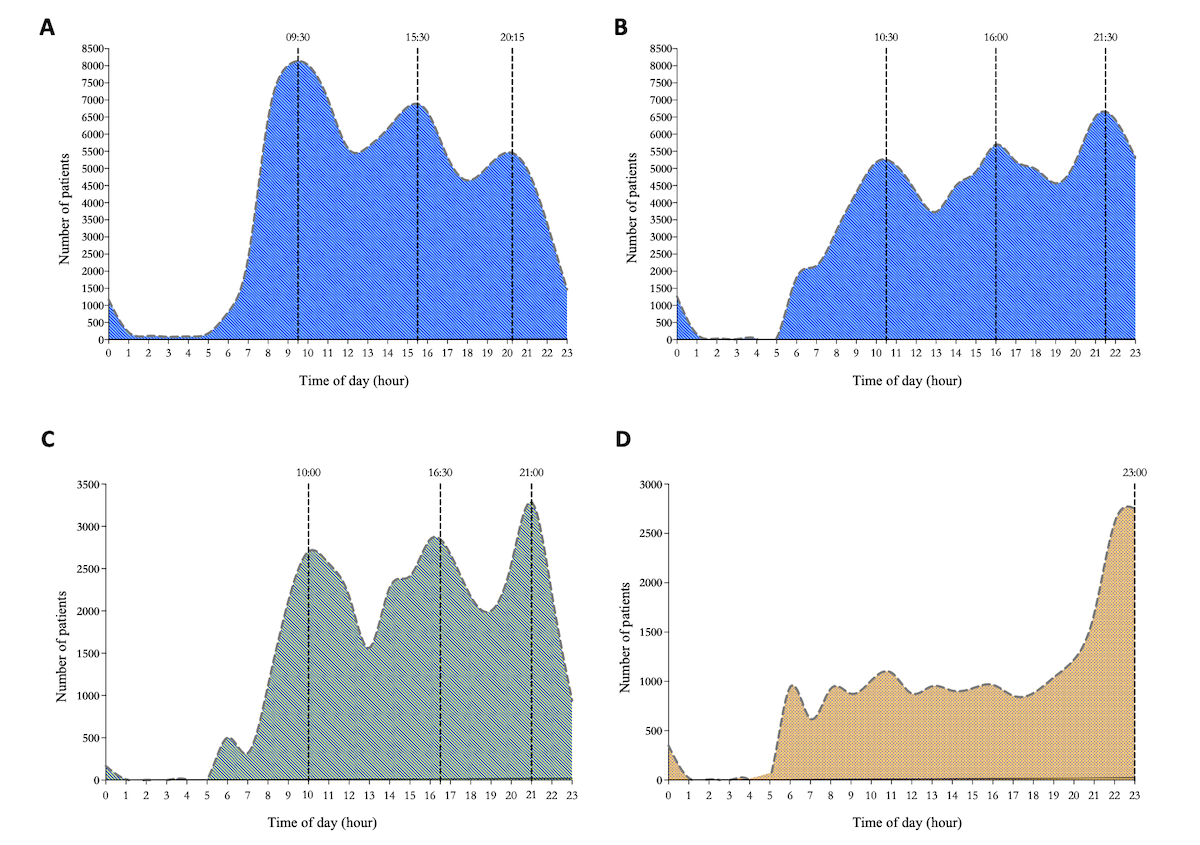
Characteristics of office and web-based doctors. (A) The time distribution of patients’ web-based visit appointments. (B) The time distribution of doctors’ web-based visit acceptances. (C)The time distribution of Dr. LJ’s web-based visit acceptances. (D) The time distribution of Dr. SA’s web-based visit acceptances.

There were 16 doctors working in the Thyroid Surgery Center of the West China Hospital. All 16 doctors offered office visits, while 13 of them also offered web-based visits. The numbers of patients seen at office and web-based visits by each doctor are shown in Multimedia Appendix 1. Doctors LJ and SA had the largest numbers of web-based patients. Of the 13 doctors who accepted web-based visits, 12 (92%) were aged 30-40 years and 1 (8%) was older than 50 years (Multimedia Appendix 1). Figure 6A and B show the times of day at which patients submitted visit applications and doctors accepted applications. Both patients’ applications and doctors’ acceptances had 3 peaks (patients’ application: 9:30 AM, 3:30 PM, and 8:15 PM; doctors’ acceptance: 10:30 AM, 4 PM, and 9:30 PM). The highest frequency of patients’ applications was at 9:30 AM, while the highest frequency of doctors’ acceptances was at 9:30 PM; this is probably because examination results usually come out at approximately 9:30 AM. Doctors are busy during the day and use their rest time at night to resume web-based consultations. Furthermore, the acceptance times of doctors LJ and SA showed different patterns, demonstrating that acceptance also depended on the daily routine of the individual doctors (Figure 6C and D). We also analyzed the time that patients spent waiting for a doctor’s reply (Multimedia Appendix 1) and found that over 50% of patients received a reply from their doctors within 300 minutes.

### Difference in Outpatient Services Between Office and Web-Based Visits

**Figure 7:**
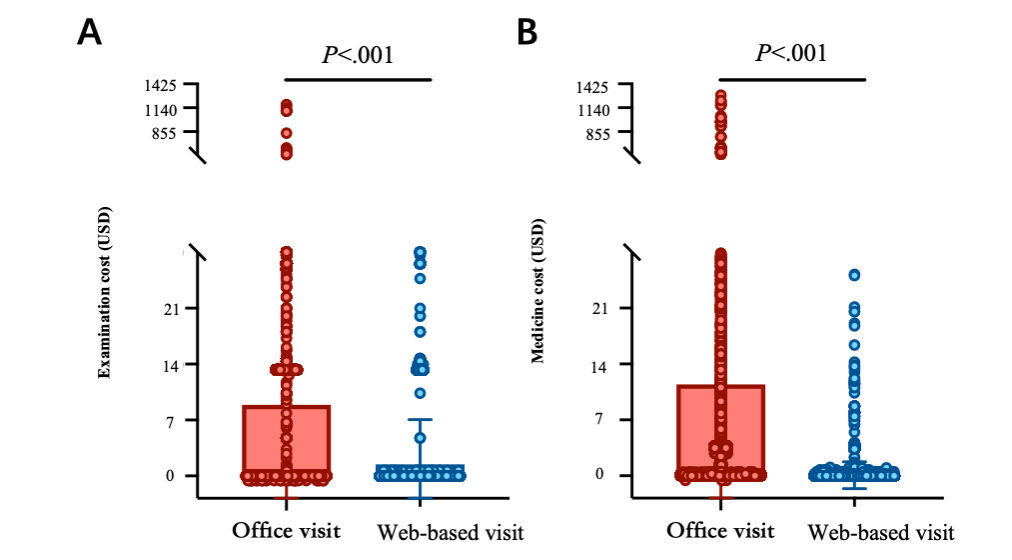
The differences between office and online visits in outpatient services.

Office and web-based visits can provide patients with medicine, examination appointments, and hospitalization registration. The medicine cost (office visit: median US $4.47, IQR 4.12-4.89; web-based visit: median US $0.96, IQR 0.88-1.03 ; *P*<.001) and examination cost (office visit: median US $5.99, IQR 5.53-6.18; web-based visit: median US $0.78, IQR 0.65-0.91; *P*<.001) of office visits were significantly higher than those of web-based visits (Figure 7A and B). This is probably because some expensive medicines and examinations can only be offered at the office, such as targeted drugs, fine needle aspiration, and contrast-enhanced ultrasound. The proportions of patients with hospitalization registration were 19,017 of 201,840 (9.5%) and 10,445 of 99,044 (10.5%) for office visits and web-based visits, respectively. This illustrated that office and web-based visits can provide the same hospitalization registration service.

## Discussion

### Principal Findings

In this study, by analyzing data from the outpatient system of the Thyroid Surgery Center in West China Hospital, we identified that web-based visits were preferred by younger patients, patients with relatively high-paying jobs, and first-visit patients. Furthermore, web-based visits may provide convenience for patients who live in remote places. We also found that the COVID-19 pandemic greatly influenced patients’ choice of outpatient service; the greater the number of confirmed COVID-19 cases, the less frequently web-based and office visit were performed. Young doctors were willing to offer web-based visits using fragments of time, which greatly saved time cost for both patients and doctors. Our study presented an overview of the thyroid outpatient service of the West China Hospital and performed multidimensional analysis of web-based visits.

### Comparison to Prior Work

In past decades, multiform types of telehealth, such as telephone-based medical consultation, have been increasingly used [28]. The COVID-19 pandemic has promoted development in the capacity to provide remote health care [29]. In this research, web-based visits constituted approximately one-third of all visits. While increased awareness of COVID-19 transmission prevention led to a return to office visits, web-based visits continued to occupy a large proportion of all visits. The number of web-based visits continued to increase while the number of office visits remained relatively stable. Chengdu and Sichuan Province experienced several epidemic outbreaks of COVID-19, during which both office and web-based visits sharply decreased. With the extension of medical payments delivered via web-based visits, the construction of telemedicine infrastructure among clinical practices, and the trend in patients’ preference for remote health care, telemedicine may become a permanent mode of health care delivery [30]. However, the long-term roles of web-based visits for patients with thyroid diseases are still vague.

Furthermore, permanent development of telehealth for routine health care calls for synchronous accessibility [10,31]. The results of this study showed that older patients, male patients, and retirees were significantly associated with a lower likelihood of choosing web-based visits. It is unsurprising that web-based visits are more difficult for patients who are inexperienced with the internet or mobile phones, and older age is related to lower internet access and lower ownership of electronic equipment such as computers and smartphones, which are necessary for web-based visits [32]. Generational differences in attitudes about internet technology, including trust of the diagnosis provided via a web-based consultation or concerns about privacy, may also influence the type of visit chosen by older patients [33]. It is worth noting that there is a high degree of acceptance of the model combining web-based and office outpatient services among patients, as almost half of patients attended both office and web-based visits. Furthermore, patients who lived outside of Sichuan were more likely to choose web-based visits because the money and time cost of web-based visits are obviously less than those of office visits. However, there was an association between outpatient visit choice and nationality. This suggested that patients in specific subgroups are likely to choose web-based visits and the barriers to choose web-based visits are not subspecialty-specific.

The doctors who offer web-based visits were mainly young doctors using fragments of time after daily work. Compared with the peak time of patients’ web-based visit application, the peak time when doctors accepted the web-based visit was delayed by 30-60 minutes. Patients were more likely to apply for web-based visits in the morning, while doctors were more likely to reply to them in the evening. The proportion of visits on the weekend were dramatically higher for web-based visits than for office visits, illustrating that web-based visits may greatly facilitate the use of weekend-time and promote medical efficiency. The doctor with the maximum number of web-based visits served more than 10,000 patients per year, which far exceeded the number of office visits. As a result, we should make efforts to encourage doctors and patients to make more use of web-based visits.

### Strengths and Limitations

Our study shows the development of web-based outpatient care in the 2 years since the outbreak of COVID-19 and is the first study to analyze the relationship between web-based outpatient care in the Thyroid Surgery Center and COVID-19 using large data volumes. There are also some limitations to this study. First, this study is a retrospective study, so we can only study the factors influencing patients’ choice of visit type rather than the effectiveness of web-based visits. Due to incomplete prospective data collection, we cannot estimate the time and money cost and satisfaction of web-based outpatient visits. In the future, we plan to carry out randomized controlled trials to supplement the research gaps in this area. Second, this study only involved thyroid diseases and did not study multiple diseases, making our findings applicable to only a limited number of diseases. We plan to extract data from multiple departments in the whole hospital and explore the similarities and differences of web-based outpatient services among various diseases. Third, at present, the services provided by our web-based visits are mainly consulting services. We need to continue to tap the potential of internet hospitals, such as for writing prescriptions and scheduling surgery appointments.

### Future Directions

To deal with existing barriers to web-based visits, we should put forward strategies to increase patients’ trust, internet knowledge, and telemedicine accessibility [34]. For older patients, targeted publicity and guidance about telemedicine services via local networks and nonelectronic methods may improve their knowledge of telemedicine [35]. Founding management systems or federal policies to ensure patients’ privacy in virtual health care, especially for consumer-facing apps, are essential to build trust among older populations [36]. Based on increased patient acceptance of telehealth, further attempts should be made to promote internet accessibility for socioeconomically disadvantaged patients [37]. Internet accessibility may also be increased through health care project coverage of telecommunication with video-based visits according to medical need.

### Conclusion

This study showed that the COVID-19 pandemic accelerated the increase in web-based visits in the Thyroid Surgery Center. Web-based visits were preferred by younger patients and patients with relatively high-paying jobs. The medicine and examination costs of office visits were significantly higher than those of web-based visits. Web-based visits exhibited higher acceptability by younger doctors.

## Appendix

Multimedia Appendix 1 Table S1 Characteristics of doctors. Table S2 The time that patients spent waiting for a doctor’s reply.

Multimedia Appendix 2 Ethics approval.

Multimedia Appendix 3 Higher resolution version of Figure 3.

Multimedia Appendix 4 STROBE checklist.

## Abbreviations

EHR: electronic health record
OR: odds ratio

## Data Availability

The data that support the findings of this study are available from each participating registry; however, restrictions apply to the availability of these data, which were used under license for the current study and are not publicly available.
