# Supplementary material for: Trends, Influence Factors, and Doctor-Patient Perspectives of Web-Based Visits for Thyroid Surgery Clinical Care: Cross-Sectional Study
Source: J Med Internet Res. 2023 Nov 7;25:e47912. doi: 10.2196/47912 (PMC10664019; doi:10.2196/47912)
Supplement: Multimedia Appendix 1 [file jmir_v25i1e47912_app1.docx]

**Table S1 Characteristics of doctors**

|  | **Age** | **Gender** | **Position** | **Office visit number** | **Online visit number** |
| --- | --- | --- | --- | --- | --- |
| **CW** | 30 | Male | Physician-in-charge | 836 | 1 |
| **GR** | 59 | Male | Chief physician | 23467 | 9 |
| **GY** | 35 | Female | Associate chief physician | 8604 | 8603 |
| **LJ** | 37 | Male | Associate chief physician | 14201 | 35733 |
| **LF** | 39 | Male | Associate chief physician | 7289 | 5231 |
| **LZ** | 53 | Male | Chief physician | 27427 | 1918 |
| **LH** | 34 | Male | Physician-in-charge | 7389 | 1911 |
| **SA** | 38 | Male | Associate chief physician | 7962 | 28978 |
| **WM** | 34 | Male | Physician-in-charge | 5353 | 1617 |
| **WX** | 39 | Male | Physician-in-charge | 12075 | 2725 |
| **WY** | 34 | Male | Physician-in-charge | 7744 | 2223 |
| **WT** | 42 | Male | Chief physician | 22116 | 7 |
| **YJ** | 35 | Male | Physician-in-charge | 15035 | 4201 |
| **ZK** | 33 | Male | Physician-in-charge | 3107 | 1239 |
| **ZW** | 31 | Female | Physician-in-charge | 1034 | 2 |
| **ZX** | 31 | Male | Physician-in-charge | 6536 | 1132 |
| **ZJ** | 65 | Male | Chief physician | 9588 | 3 |
| **ZXH** | 36 | Male | Associate chief physician | 13491 | 2852 |

**Table S2 The time that patients spent waiting for a doctor’s reply.**

| Waiting time (min) | Number of visit (patient) |
| --- | --- |
| 1-100 | 25185 |
| 101-200 | 18567 |
| 201-300 | 7929 |
| 301-400 | 4558 |
| 401-500 | 3677 |
| 501-600 | 3314 |
| 601-700 | 2960 |
| 701-800 | 2691 |
| 801-900 | 2437 |
| 901-1000 | 1770 |
| 1001-1100 | 1586 |
| 1101-1200 | 1627 |
| 1201-1300 | 2005 |
| 1301-1400 | 2512 |
| 1401-1500 | 3161 |
| >1500 | 633 |
