# Supplementary figures and images for: Trends, Influence Factors, and Doctor-Patient Perspectives of Web-Based Visits for Thyroid Surgery Clinical Care: Cross-Sectional Study
Source: J Med Internet Res. 2023 Nov 7;25:e47912. doi: 10.2196/47912 (PMC10664019; doi:10.2196/47912)

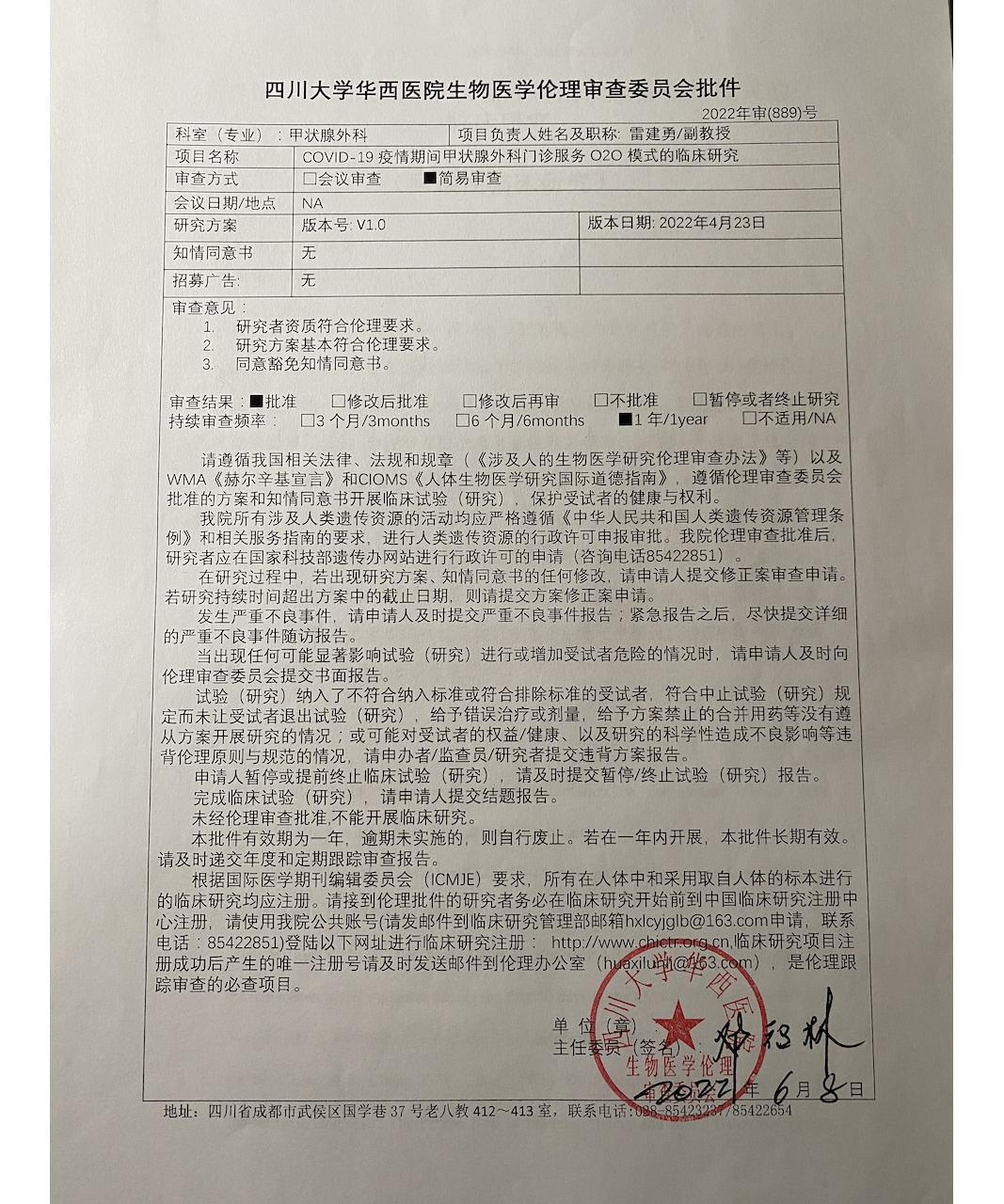

Supplement: Multimedia Appendix 2 [file jmir_v25i1e47912_app2.png]

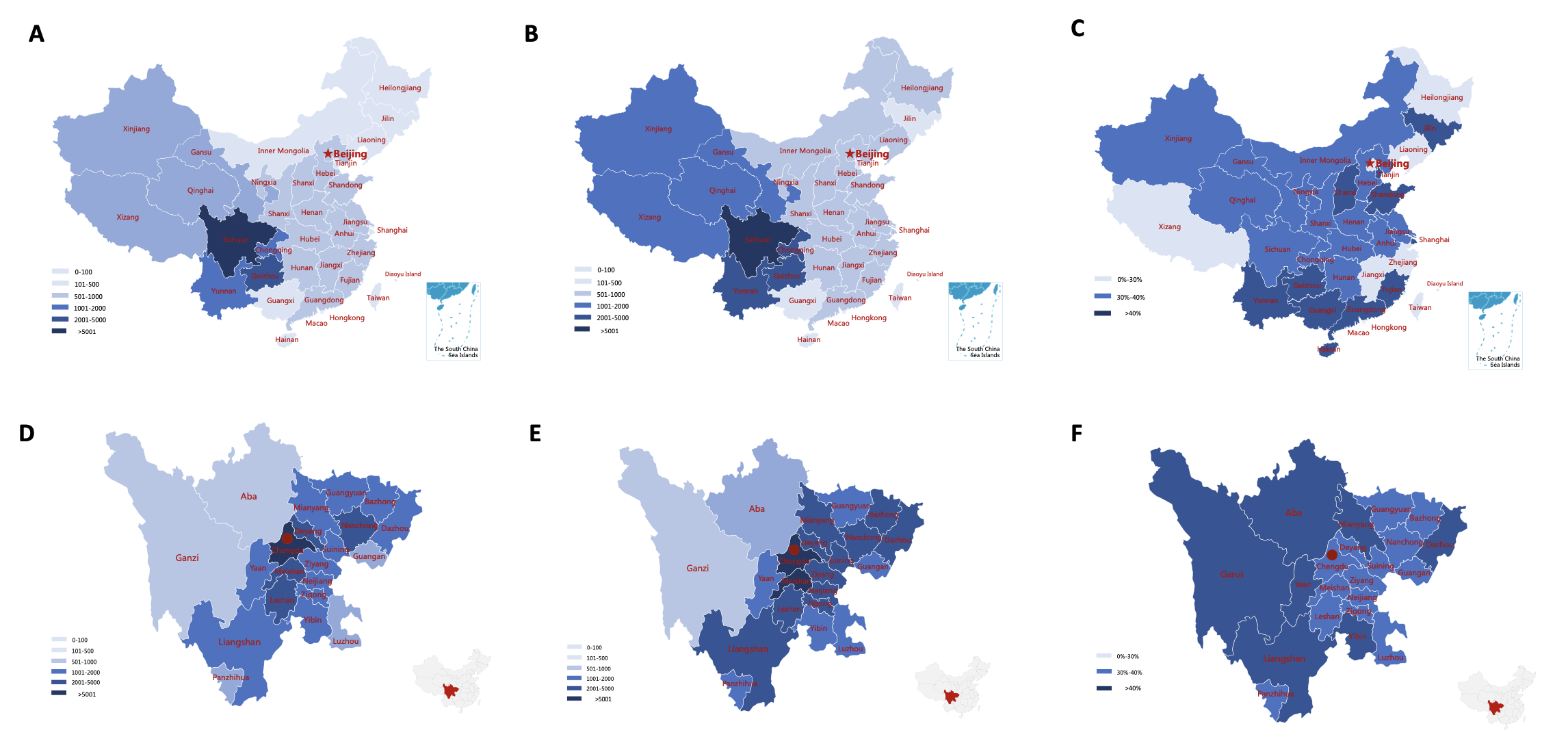

Supplement: Multimedia Appendix 3 [file jmir_v25i1e47912_app3.png]
