# Supplementary material for: Trends, Influence Factors, and Doctor-Patient Perspectives of Web-Based Visits for Thyroid Surgery Clinical Care: Cross-Sectional Study
Source: J Med Internet Res. 2023 Nov 7;25:e47912. doi: 10.2196/47912 (PMC10664019; doi:10.2196/47912)
Supplement: Multimedia Appendix 4 [file jmir_v25i1e47912_app4.pdf]

STROBE checklist

Section and Item	Item No.	Recommendation	Page	Section in manuscript
Title and abstract	1 (a)	Indicate the study's design with a commonly used term in the title or the abstract	1	Title page
	(b)	Provide in the abstract an informative and balanced summary of what was done and what was found	1-2	Abstract
Introduction				
Background/rationale	2	Explain the scientific background and rationale for the investigation being reported	2	Introduction
Objectives	3	State specific objectives, including any prespecified hypotheses	4	Introduction
Methods				
Study design	4	Present key elements of study design early in the paper	4	Online and office visit process
Setting	5	Describe the setting, locations, and relevant	4	Data source

		dates, including periods of recruitment, exposure, follow-up, and data collection		
Participants	6	Cohort study—Give the eligibility criteria, and the sources and methods of selection of participants. Describe methods of follow-up.	5	The socioeconomic and demographic information of patients; The information of doctors
Variables	7	Clearly define all outcomes, exposures, predictors, potential confounders, and effect modifiers. Give diagnostic criteria, if applicable	6	Identification of factors influencing the choice of visit type
Data sources/ measurement	8*	For each variable of interest, give sources of data and details of methods of assessment (measurement). Describe comparability of assessment methods if there	4	Data source

		is more than one group		
Bias	9	Describe any efforts to address potential sources of bias	6	Statistical Analysis
Study size	10	Explain how the study size was arrived at	5	The socioeconomic and demographic information of patients; The information of doctors
Quantitative variables	11	Explain how quantitative variables were handled in the analyses. If applicable, describe which groupings were chosen and why	5	The socioeconomic and demographic information of patients; The information of doctors
Statistical methods	12	(a) Describe all statistical methods, including those used to control for confounding<br>(b) Describe any methods used to examine subgroups and interactions	6	Statistical Analysis

		(c) Explain how missing data were addressed<br>(d) Cohort study—If applicable, explain how loss to follow-up was addressed<br>(e) Describe any sensitivity analyses		
Results				
Participants	13*	(a) Report numbers of individuals at each stage of study—eg numbers potentially eligible, examined for eligibility, confirmed eligible, included in the study, completing follow-up, and analyzed<br>(b) Give reasons for non-participation at each stage<br>(c) Consider use of a flow diagram	6-8	The socioeconomic characteristics of office and online outpatient services<br><br>Table 1, Table S1
Descriptive data	14*	(a) Give characteristics of study participants (eg demographic, clinical, social) and information	6-9	The socioeconomic characteristics of office and online

		on exposures and potential confounders<br>(b) Indicate number of participants with missing data for each variable of interest<br>(c) Cohort study—Summarise follow-up time (eg, average and total amount)		outpatient services<br><br>The demographic characteristics of office and online outpatient services<br><br>Table 1, Table s1
Outcome data	15*	Cohort study—Report numbers of outcome events or summary measures over time<br>Case-control study—Report numbers in each exposure category, or summary measures of exposure<br>Cross-sectional study—Report numbers of outcome events or summary measures	9-11	The increase in online visits and its relationship with the COVID-19 pandemic<br><br>Factors influencing the choice of visit type
Main results	16	(a) Give unadjusted estimates and, if applicable, confounder-	9-11	Factors influencing the choice of visit type

		adjusted estimates and their precision (eg, 95% confidence interval). Make clear which confounders were adjusted for and why they were included<br>(b) Report category boundaries when continuous variables were categorized<br>(c) If relevant, consider translating estimates of relative risk into absolute risk for a meaningful time period		The characteristics of doctors providing outpatient services<br>The difference in outpatient services between office and online visits
Other analyses	17	Report other analyses done—eg analyses of subgroups and interactions, and sensitivity analyses	N/A	N/A
Discussion				
Key results	18	Summarise key results with reference to study objectives	11	Principal Findings
Limitations	19	Discuss limitations of the study, taking into account sources of potential bias or	13-14	Strengths and Limitations

		imprecision. Discuss both direction and magnitude of any potential bias		
Interpretation	20	Give a cautious overall interpretation of results considering objectives, limitations, multiplicity of analyses, results from similar studies, and other relevant evidence	13-14	Comparison to Prior Work
Generalisability	21	Discuss the generalisability (external validity) of the study results	13-14	Strengths and Limitations
Other information				
Funding	22	Give the source of funding and the role of the funders for the present study and, if applicable, for the original study on which the present article is based	16	Funding statement
